# Discovery of a *Manduca sexta* Allatotropin Antagonist from a *Manduca sexta* Allatotropin Receptor Homology Model

**DOI:** 10.3390/molecules23040817

**Published:** 2018-04-03

**Authors:** Zhen-Peng Kai, Jing-Jing Zhu, Xi-Le Deng, Xin-Ling Yang, Shan-Shan Chen

**Affiliations:** 1 School of Chemical and Environmental Engineering, Shanghai Institute of Technology, Shanghai 201418, China; kaizp@sit.edu.cn (Z.-P.K.); 15212123252@163.com (J.-J.Z.); 2 Institute of Agro-Food Standards and Testing Technologies, Shanghai Academy of agricultural Science, Shanghai 201403, China; 3 Department of Applied Chemistry, College of Science, China Agricultural University, Beijing 100193, China; chemdxl@gmail.com

**Keywords:** allatotropin, insecticide, GPCR, antagonist

## Abstract

Insect G protein coupled receptors (GPCRs) have important roles in modulating biology, physiology and behavior. They have been identified as candidate targets for next-generation insecticides, yet these targets have been relatively poorly exploited for insect control. In this study, we present a pipeline of novel *Manduca sexta* allatotropin (Manse-AT) antagonist discovery with homology modeling, docking, molecular dynamics simulation and structure-activity relationship. A series of truncated and alanine-replacement analogs of Manse-AT were assayed for the stimulation of juvenile hormone biosynthesis. The minimum sequence required to retain potent biological activity is the *C*-terminal amidated octapeptide Manse-AT (6–13). We identified three residues essential for bioactivity (Thr^4^, Arg6 and Phe^8^) by assaying alanine-replacement analogs of Manse-AT (6–13). Alanine replacement of other residues resulted in reduced potency but bioactivity was retained. The 3D structure of the receptor (Manse-ATR) was built and the binding pocket was identified. The binding affinities of all the analogs were estimated by calculating the free energy of binding. The calculated binding affinities corresponded to the biological activities of the analogs, which supporting our localization of the binding pocket. Then, based on the docking and molecular dynamics studies of Manse-AT (10–13), we described it can act as a potent Manse-AT antagonist. The antagonistic effect on JH biosynthesis of Manse-AT (10–13) validated our hypothesis. The IC_50_ value of antagonist Manse-AT (10–13) is 0.9 nM. The structure-activity relationship of antagonist Manse-AT (10–13) was also studied for the further purpose of investigating theoretically the structure factors influencing activity. These data will be useful for the design of new Manse-AT agonist and antagonist as potential pest control agents.

## 1. Introduction

Insect neuropeptides control the most critical metabolic, homeostatic, developmental, reproductive and behavioural events during an insect’s life. They may function both as hormones and neurotransmitters or neuromodulators, mainly mediated by the interaction with G protein coupled receptors (GPCRs). The commercial agricultural community has an interest in safe insecticides that are selective in their mechanisms of action, environmentally friendly and discriminative only against target species. With this in mind, research into the application of insect neuropeptides for pest control has been closely monitored and even promoted by the appropriate industries. Some insect neuropeptides with short chains would appear paramount candidates [1]. The rational design of agonists or antagonists of neuropeptides that affects receptor interaction is indicated as a general strategy concept for the use of insect neuropeptides for pest control, but, up to now, there no commercial insecticides have been discovered using insect neuropeptides as the lead compounds or with their receptors (GPCRs) as the targets. Some reasons are the facts that the mechanisms of their biological activities and the knowledge of the receptor–neuropeptide complex three-dimensional (3D) structure are far from being fully characterized or understood. One way to get a better insight into the mode of activity and the functional diversity of insect neuropeptides is by the use of receptor-selective agonists and antagonists. Despite the intensive studies of insect neuropeptides antagonists, only a few antagonists have been discovered to date, mainly due to the lack of defined methods for obtaining antagonists on the basis of a known neuropeptide agonist, and because of the inability to predict which conformation will lead to a highly potent inhibitory or stimulatory receptor-selective activity. In the past few years, some insect neuropeptides antagonists were found mainly through a random observation. Nachman et al. found analog **4b** at 10 mM partially antagonized the juvenile hormone (JH) biosynthesis inhibitory properties of a 10 nM solution of Dippu-AST5 when they did the studies of allatostatin analogues incorporating turn-promoting moieties [2]. Scientists also developed some rational approaches to design the antagonists. Altstein and his cooperators have developed a novel integrated approach termed backbone cyclic neuropeptide based antagonist (BBC-NBA). They applied this approach to the insect pyrokinin (PK)/pheromone biosynthesis activating neuropeptide (PBAN) family, and resulted in the discovery of highly potent, stable, and selective antagonists [3].

*Manduca sexta* allatotropin (Manse-AT) is an amidated tridecapeptide that was isolated from pharate adult heads based on its ability to stimulate JH biosynthesis by the corpora allata (CA) of adult female lepidopteran insects in vitro [4]. Manse-AT is a member of a large family of structurally-related peptides which are widely found in insects and also in other invertebrates that lack JH [5]. These peptides also exhibit myotropic activities in numerous invertebrate species which led to the suggestion that the original role for this family of peptides is myotropic, and stimulation of JH biosynthesis arose secondarily in insects. Other biological functions have been described which include the cardioacceleratory activity on the adult heart in lepidopteran insects and the inhibition of active ion transport across the larval midgut epithelium in *M. sexta* [6,7]. It has been shown that the *C*-terminal amide of Manse-AT is essential for full biological activity on the CA [8]. Truncation of the *N*-terminal 5 amino acids of Manse-AT had little effect on bioactivity, but further truncation resulted in greatly reduced activity. It indicated that the amidated peptide Manse-AT (6–13) is the core peptide essential for bioactivity on the CA. The relative potencies of these analogs were not determined however.

Horodyski et al. isolated a cDNA for AT receptor from *M. sexta* (Manse-ATR) by a PCR-based approach using the cDNA sequence of Bommo-ATR [9]. The sequence of Manse-ATR is most similar to Bommo-ATR, and to several GPCRs from other insect species, including *A. aegypti* [10], and *T. castaneum* [11], which have been functionally characterized as AT receptors.

In this study, we used Manse-AT (6–13), which contains the active core of Manse-AT, and quantified the biological activities of a series of truncated and alanine-replacement Manse-AT (6–13) analogs to confirm the identity of the active core and determine the residues most critical for the stimulation of JH biosynthesis by the adult female CA. We built the 3D structure of Manse-ATR by homology modeling using the crystal structure of the nociceptin/orphanin FQ receptor as the template and identified the ligand-binding pocket using blind docking calculations. The binding affinities of all the analogs were estimated by calculating the free energy of binding. The calculated binding affinities correlate with the relative potencies of these peptide analogs in biological assays using the CA. This is an important first step in understanding the structural basis of ligand binding to Manse-ATR and will aid in the development of agonists and antagonists. Then, based on the docking and molecular dynamics studies of Manse-AT (6–13) analogs, we described Manse-AT (10–13) acts as a potent Manse-AT antagonist. The structure-activity relationship (SAR) of antagonist Manse-AT (10–13) was also studied in this paper.

## 2. Results and Discussion

### 2.1. Effects of Manse-AT and Analogs on JH Biosynthesis

The ability of Manse-AT and analogs stimulate JH biosynthesis was evaluated in vitro. The EC_50_ value of each peptide was shown in Table 1. The Dose response curves of Manse-AT was shown in Supplementary Materials, Figure S1. The truncated peptide Manse-AT (6–13) stimulated JH biosynthesis with an EC_50_ value of 18.4 nM, which is about 20-fold less potent than Manse-AT (EC_50_ 0.9 nM). The stimulation of JH biosynthesis of Manse-AT (6–13) at 1 μM (a high concentration for a hormone) is 600%, which is similar to the stimulation of natural peptide Manse-AT. Statistical analyses of a Dunnett’s multiple comparison test following one-way ANOVA show there are no significant differences between Manse-AT (6–13) and natural peptide Manse-AT both on the EC_50_ values and stimulation of JH biosynthesis at a high concentration (Supplementary Materials, Figure S2). This analog was previously shown to possess strong bioactivity, but no dose-response or quantitative comparison with Manse-AT has been shown until now [4]. We then synthesized a series of analogs truncated from the amino terminus of Manse-AT (6–13). Assays of these analogs show that the heptapeptide Manse-AT (7–13) also had a strong effect on JH biosynthesis (EC_50_ 179.8 nM), but was 200-fold less potent compared with Manse-AT (Table 1), and the stimulation at 1 μM is 232.9%, which is about half the potency of Manse-AT. Statistical analyses between Manse-AT (7–13) and Manse-AT on their EC_50_ values shows a significant difference with P value less than 0.05 (Supplementary Materials, Figure S2). Further truncations rapidly abolished bioactivity such that any effect was only demonstrated at very high, non-physiological concentrations. The EC_50_ value of Manse-AT (9–13), Manse-AT (10–13) and Manse-AT (11–13) cannot be calculated because of the low effect at each concentration. Meanwhile the stimulation at 1 μM of Manse-AT (9–13), Manse-AT (10–13) and Manse-AT (11–13) have no different from ZERO based on t-test (data not shown). The bioassays result of truncated Manse-AT analogs suggests that Manse-AT (6–13) can be a good lead compound in the structure-activity studies.

An analog series for Manse-AT (6–13) was synthesized to determine the importance of each peptide side chain for the stimulation of JH biosynthesis by adult female CA in vitro. Alanine was chosen as the replacement amino acid for the analog series and their potencies were shown in Table 1. Manse-AT (6–13) [Ala^6^] and Manse-AT (6–13) [Ala^8^] were completely inactive, even at a concentration of 10 µM. Manse-AT (6–13) [Ala^4^] (EC_50_: 8958 nM) exhibited activity, but only at very high, pharmacological concentrations. Alanine replacement of amino acids in the *N*-terminal tripeptide resulted in analogs that exhibited bioactivity, but only at relatively high concentrations. Their stimulations at 1 μM are less than half the potency of Manse-AT (6–13). Statistical analyses shows only Manse-AT (6–13) [Ala^7^] has no significant difference from Manse-AT (6–13) in Supplementary Materials, Figure S3. The conservative (Ala for Gly) substitution in Manse-AT (6–13) [Ala^7^] had little effect on bioactivity. The t-test show Manse-AT (6–13) [Ala^4^], Manse-AT (6–13) [Ala^6^] and Manse-AT (6–13) [Ala^8^] are no different from ZERO (data not shown). It suggested that Thr^4^, Arg^6^ and Phe^8^ of Manse-AT (6–13) are the most important residues for JH biosynthesis. This SAR on JH biosynthesis is similar to that on the inhibition of active ion transport across the larval midgut epithelium of *M. sexta* [8].

### 2.2. Manse-ATR Model

Because of the difficulties in crystallizing membrane proteins, homology modeling of GPCR 3D structures is a useful method for the study of the interaction of GPCRs with their ligands [12]. Recently, there has been a great deal of progress in solving the structures of GPCRs, and several new crystal structures have been reported. We identified the nociceptin/orphanin FQ receptor (PDB ID: 4EA3) [13], which not only has a high homology with Manse-ATR (28% identical and 48% similar for over the entire length of the receptor), but also contains a peptide mimetic as the ligand. Sequence identities of the 7TM helices and loops of Manse-ATR with those of the nociceptin/orphanin FQ receptor were also calculated. The 7TM helices are 75%, 57%, 71%, 44%, 47%, 50%, 55% identical between the two receptors, respectively, and the three extracellular loops (ECLs) are 67%, 58%, and 50% identical, respectively. Each 7TMs and ECLs has a high identity with those of the nociceptin/orphanin FQ receptor. Thus, the crystal structure of the nociceptin/orphanin FQ receptor containing a peptide mimetic was selected as the template to build the structure of Manse-ATR in this study.

Five models of Manse-ATR were generated using the FUGUE and ORCHESTRAR modules in Sybyl-X 2.0. The initial models were optimized energetically using the minimize program with steepest descent algorithm, AMBER7 FF99 as the force field and Gasteiger-Huckel as the atomic point charges. The minimization was terminated when the RMS gradient convergence criterion of 0.05 kcal/(mol·Å) was reached. In order to select the best model, we checked the structural validity of Manse-ATR by PROCHECK. The geometry of the final refined model was evaluated with Ramachandran’s plot calculation computed with the PROCHECK program. The torsion angles of *φ* and *ψ* in the generated models are represented in the Ramachandran plot as shown in Figure S4 (Supplementary Materials). These torsion angles of 88.4% of the residues had values within the most favored regions and only 0.3% of the residues had values within disallowed regions and the overall G-factor [14] is 0.26. The overall G-factor is a measure of the overall normality of the structure and low G-factors indicate that residues have likely conformations. In the Ramachandran plot, the stereochemical quality of a protein model can be judged by the use of *φ*, *ψ* scatter plots, with incorrect structures generally having a much larger fraction of residues lying in disallowed regions. Since our model of Manse-ATR has only 0.3% of its residues in disallowed regions, we conclude that our structure satisfies the criteria of a good homology model.

We have simulated for 10 ns the Manse-ATR model to obtain a stable and low energy conformation using molecular dynamics. By reporting the root mean square deviation (RMSD) of the protein structure from the starting model, the receptor differentiates in structure and reaches a relative stable conformational minimum after approximately 3 ns. The total energy (E_tot_) and potential energy (E_p_) of the system are constant during the molecular dynamics simulation. The conformation with the lowest energy of the final 100 structures from the MD simulation was selected as the final structure of Manse-ATR.

**Figure 1:**
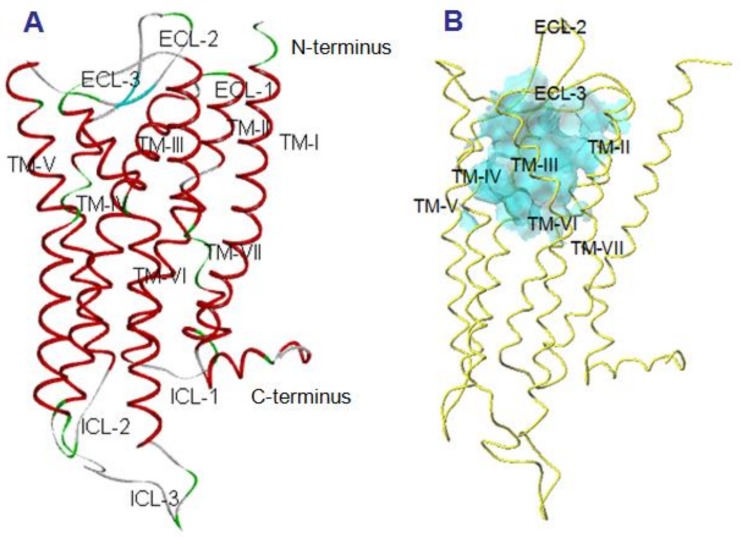
The 3D structure of Manse-ATR. (**A**) The seven transmembrane alpha helices (TM-I–TM-VII) are displayed in red, and are connected by the extracellular (ECL) and intracellular (ICL) loops. There is a β-strand in the ECL2 (cyan), and a α-helix in the *C*-terminus (red); (**B**) The probable ligand binding pocket of Manse-ATR. Side view of the binding pocket is displayed as a cyan transparent surface. There is a large hydrophobic pocket in the extracellular portion of the Manse-ATR model (consisting of TM helices and extracellular loops). This is the probable binding site of Manse-AT and the analogs used in this study.

The final 3D structure of Manse-ATR is presented in Figure 1A. There is a large hydrophobic pocket displayed as a cyan transparent surface in the extracellular part of Manse-ATR model, which is the probable binding site with Manse-AT analogs (Figure 1B). This hydrophobic pocket was selected as the candidate binding site for the identification of the binding pocket using the docking calculations.

### 2.3. The Binding Pocket in Manse-ATR

**Figure 2:**
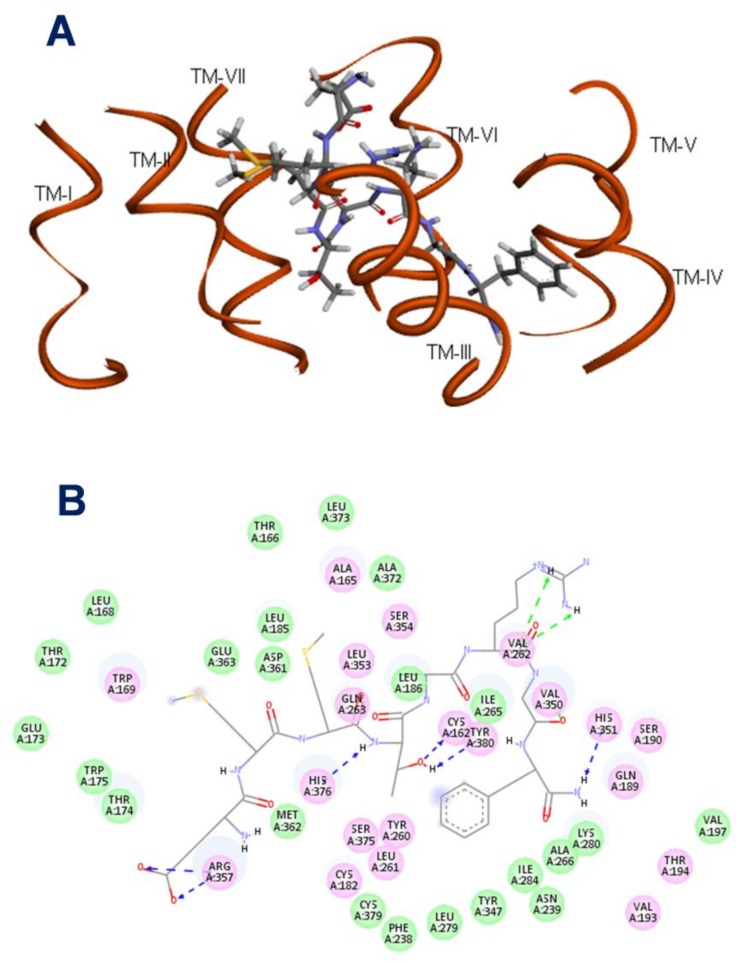
Predicted structure of Manse-ATR binding pocket with Manse-AT (6–13) as ligand. (**A**) Manse-AT (6–13) occupying the binding pocket of Manse-ATR. The putative binding pocket of Manse-ATR was localized between helices II, III, IV, V, VI and VII, ECL2 and ECL3; (**B**) the important residues of the binding pocket. Cys162 (TM-II), Val262 (ECL-2), His351 (TM-VI), Arg357 (TM-VI), His376 (TM-VII) and Tyr380 (TM-VII) of receptor have hydrogen bonding interactions with the residues Glu1, Thr^4^, Arg^6^ and *C*-terminal amide of Manse-AT (6–13). The Phe^8^ of Manse-AT (6–13) has a strong hydrophobic interaction with the pocket. This analysis was done by Discovery Studio 3.5 Visualizer.

To investigate the Manse-ATR model further, blind docking calculations of the active analog, Manse-AT (6–13), was used to identify a receptor binding pocket and to analyze the binding characteristics of the analogs used in this study. A putative binding pocket of Manse-ATR (Figure 2) was found between helices II, III, IV, V, VI and VII, ECL2 and ECL3. Figure 2B shows the localization of the important residues of the binding pocket. Hydrogen bonding interactions show that Manse-AT (6–13) is bound to Cys162 (TM-II), Val262 (ECL-2), His351 (TM-VI), Arg357 (TM-VI), His376 (TM-VII) and Tyr380 (TM-VII) of Manse-ATR. The Glu1, Thr^4^, Arg^6^ residues and *C*-terminal amide of Manse-AT (6–13) form hydrogen bonds with the above residues of the binding pocket, and the Phe^8^ of Manse-AT (6–13) has a strong hydrophobic interaction with the binding pocket (Figure 2B). The results of alanine-replacement of Manse-AT (6–13) show that Thr^4^, Arg^6^ and Phe^8^ are critical for full biological activity. Amidation of the carboxy terminus is also essential for full biological activity [8]. The results of our biological assays confirm that the essential residues for bioactivity make significant contacts within the binding pocket of Manse-ATR. The shortest peptide with full biological activity consists of amino acids 6–13 [Manse-AT (6–13)] (Table 1). The hydrogen bond between Glu1 of Manse-AT (6–13) and Arg357 of Manse-ATR can explain why the biological activity is lost upon further truncation of the peptide. 

### 2.4. Binding Affinities of Manse-AT Analogs

**Figure 3:**
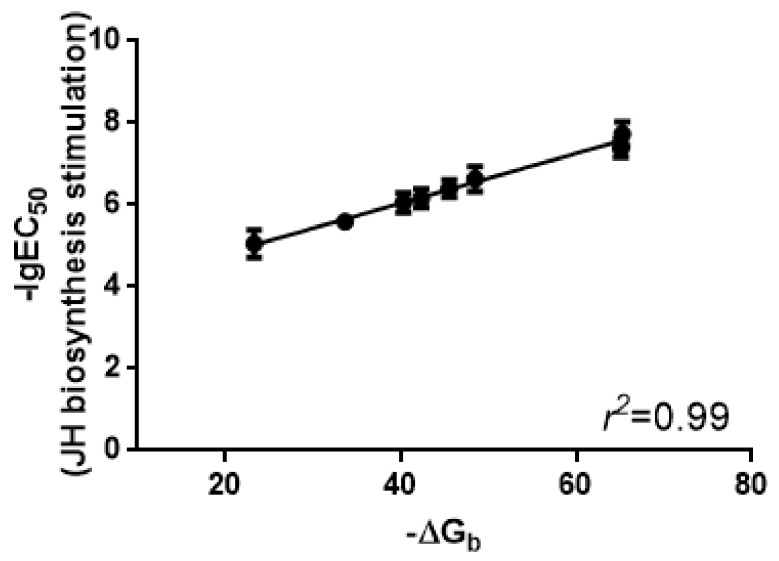
The estimated free energy of binding (∆*G*_b_ values) versus the EC_50_ values for the stimulation of JH biosynthesis by the adult female CA.

This binding pocket was used in the study of binding affinities of Manse-AT analogs to validate the binding pocket and our Manse-ATR model. The estimated free energy of binding (∆*G*_b_) is an indication of the binding affinity of a ligand to its receptor. Each of the Manse-AT (6–13) analogs containing the truncated and Ala-replacements were docked into the binding pocket, and their ∆*G*_b_ values were calculated (Table 1). A ligand can be docked into the receptor spontaneously, only when the free energy of binding is negative. When the free energy is lower, the binding affinity will be stronger, and the corresponding bioactivity is predicted to be greater. Manse-AT (10–13), Manse-AT (11–13), Manse-AT (6–13) [Ala^6^] and Manse-AT (6–13) [Ala^8^] which possess a positive free energy of binding, are completely inactive in our bioassays. To validate our prediction of the binding pocket and the Manse-ATR model, the linear regressions between free energy of binding and the experimental bioactivity values were calculated. There is an excellent linear correlation between the value of estimated free energy of binding versus experimental EC_50_ values for the stimulation of JH biosynthesis (*r*^2^ value of 0.99 and *F* value of 463.8) (Figure 3). These data confirms the validity of our Manse-ATR model and the prediction of the ligand binding pocket. The discovery of new Manse-AT antagonist was further performed using this Manse-ATR model in this study.

### 2.5. Antagonist Design

**Figure 4:**
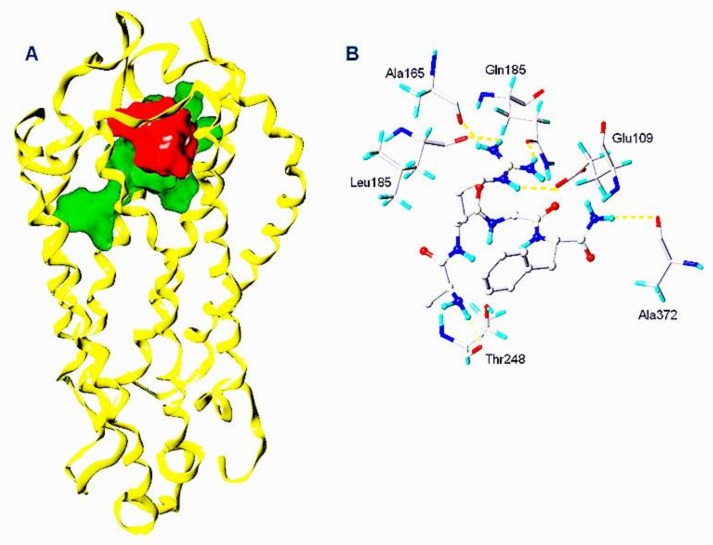
Predicted structure of Manse-AT (10–13) binding pocket. (**A**) Comparison of binding pockets of Manse-AT (6–13) (shown in green) and Manse-AT (10–13) (shown in red); (**B**) The hydrogen bond interactions of Manse-AT (10–13) with Manse-ATR. Hydrogen bonds were shown in yellow.

In the study of binding affinities, Manse-AT (10–13) and Manse-AT (11–13) showed poor interactions with the bonding pocket of Manse-AT (6–13). Further blind docking calculations of Manse-AT (10–13) and Manse-AT (11–13) were used to identify different receptor binding pocket in this study. The ∆*G*_b_ value of Manse-AT (10–13) is 3.23 kJ/mol when it was docked into the same binding pocket of Manse-AT (6–13), but it can be docked into a different binding pocket of Manse-ATR (shown in Figure 4) with a negative ∆*G*_b_ value, −84.37 kJ/mol. Figure 4A showed the binding pocket of Manse-AT (10–13) (shown in red) was occupying the upper side of the binding pocket of Manse-AT (6–13) (shown in green). Manse-AT (10–13) formed six hydrogen bonds with Glu 109 (*N*-terminus), Ala 165 (TM-II), Leu 185 (TM-III), Gln 189 (TM-III), Thr 248 (ECL-2) and Ala 372 (TM-VII) of this binding pocket (Figure 4B). The guanidinium group of the residue Arg, *C*-terminal amide and *N*-terminal amide of Manse-AT (10–13) form hydrogen bonds with the above residues of the binding pocket. The binding affinity of Manse-AT (10–13) interacted with Manse-AT (10–13) binding pocket (∆*G*_b_ value: −84.37 kJ/mol) is stronger than that of Manse-AT (6–13) interacted with Manse-AT (6–13) binding pocket (∆*G*_b_ value: −65.23 kJ/mol). The binding process of Manse-AT to the receptor can be described as the *C*-terminal tetrapeptide was bound into the binding pocket of the receptor, then the *N*-terminal changes the conformation of ligand-receptor complex into the active conformation and the G-protein was released because of the conformation change. Manse-AT (10–13) has a potent binding affinity with the upper side of the pocket of Manse-ATR, which suggested that Manse-AT (10–13) might act as a blocker of Manse-AT during its docking procedure. The similar study of Manse-AT (11–13) was performed, but no binding pocket of Manse-AT (11–13) was found.

To validate the binding pocket of Manse-AT (10–13) that we found above and study structure activity relationship (SAR), twelve Manse-AT (10–13) analogs were designed (Table 2). In analogs **1** to **4**, the *N*-terminal residue Ala was replaced with Gly, Leu, isobutyric acid and (9*H*-fluoren-9-yl) methyl hydrogen carbonate (Fmoc). The Arg residue was replaced with Ala and Leu in analogs **5** and **6**, respectively. Ala and cyclopropylalanine (Cpa) replaced the Gly residue of Manse-AT (10–13) in analogs **7** and **8**. In analogs **9** to **12**, the *C*-terminal residue Phe was replaced with Ala, Tyr, 4-methyl-l-phenylalanine and 4-chloro-l-phenylalanine, respectively.

### 2.6. Antagonistic Effects on JH Biosynthesis

**Figure 5:**
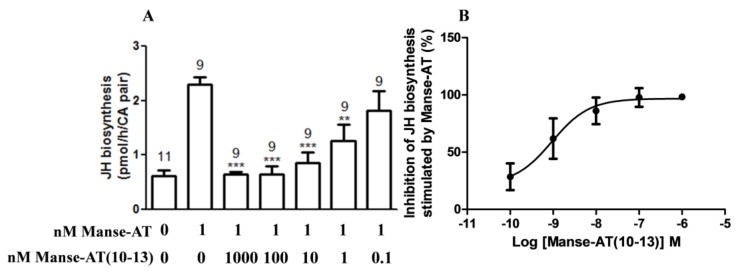
Antagonistic effects of Manse-AT (10–13) on JH biosynthesis. (**A**) Manse-AT (10–13) is a potent Manse-AT antagonist in the CA; (**B**) Dose response curves of Manse-AT (10–13) on the inhibition of JH stimulation of Manse-AT. Each bar represents the mean ± SEM for the number of individual measurements indicated error bars. Asterisks indicate significant differences between Manse-AT and mixture solutions as determined by Dunnett’s multiple comparison test following one-way ANOVA: ** 0.0001 < *p* < 0.05; *** *p* < 0.0001.

Manse-AT (10–13) has a potent ability to inhibit the stimulation of Manse-AT on the JH biosynthesis (Figure 5A). The IC_50_ value of antagonist Manse-AT (10–13) is 0.9 nM, which equals the EC_50_ value of Manse-AT on the JH biosynthesis (Figure 5B). Manse-AT (11–13) did not possess any antagonistic effect to Manse-AT.

The ability of all Manse-AT (10–13) analogs to inhibit the stimulation of Manse-AT on JH biosynthesis was evaluated in vitro, shown in Table 2. In our work, we chose organic acids or hydrophobic amino acids to mimic the residue Ala of Manse-AT (10–13) to design analog **1** to **4**. In comparison to the lead Manse-AT (10–13), these analogs showed different antagonistic effects on JH biosynthesis. Analog **1**, in which the *N*-terminal Ala of the lead replaced with Gly, had the same IC_50_ value as that of the lead peptide. The bioactivity of **2** was almost 40-fold less than that of the lead, probably because the side chain of Leu is larger than Ala. In analog **4**, the Ala was replaced with a large hydrophobic group of Fmoc. Its IC_50_ value could not be calculated because it showed no effect. It indicates that the free space of the *N*-terminal Ala residue should remain small. If some bigger organic groups are used to replace Ala, the analog should be inactive or less active. Figure 4B showed the *N*-terminal amide of Manse-AT (10–13) forms a hydrogen bond with the binding pocket. To validate this model, analog **3** was designed, in which the *N*-terminal Ala was mimicked by isobutyric acid. The IC_50_ value of **3** was almost 30-fold less than that of the lead. It indicates that this hydrogen bond interaction is critical to the antagonistic effect. In analogs **5** and **6**, the Arg was replaced with Ala and Leu, respectively, in which residues are hydrophobic organic groups. These analogs have no antagonistic effect to Manse-AT on JH biosynthesis. The IC_50_ values of those analogs suggest that the guanidinium group of the Arg residue is critical to bioactivity and its position must be considered carefully in the design of analogs. These data also confirms the validity of the prediction of the Manse-AT (10–13) binding pocket. Ala and Cpa was chosen to mimic the residue Gly of Manse-AT (10–13) to design analog **7** and **8**. The IC_50_ value of **7** is 20-fold less than that of the lead peptide, whereas the bioactivity of **8** is almost 1145-fold less than that of Manse-AT (10–13). The free space of the Gly residue should also remain small. If some bigger organic groups, such as Ala or Cpa are used to replace Gly, the analog should be less active. The IC_50_ value of **10**, **11** and **12** is similar to that of the lead, whereas analog **9** is inactive, compared with Manse-AT (10–13). The significant activity of **10**, **11** and **12**, suggests that the *C*-terminal Phe could be substituted in analog design, and the presence of an aromatic group could help to retain the significant activity.

## 3. Materials and Methods

### 3.1. Chemicals

Manse-AT, citronellol, thioanisole, dithioglycol, phenol, HPLC grade *n*-hexane, *N*,*N*-dimethyl-formamide (DMF), dichloromethane (DCM) and acetonitrile were from Sigma-Aldrich (St. Louis, MO, USA). Rink Amide-AM resin (0.52 mmol/g substitution), *O*-benzotriazole-*N*,*N*,*N*′,*N*′-tetramethyl-uronium-hexafluoro-phosphate (HOBt), 1-hydroxybenzotriazole anhydrate (HBTU), *N*,*N*′-diisopropyl ethylamine (DIEA), trifluoroacetic acid (TFA) and Fmoc-protected amino acids were purchased from GL Biochem (Shanghai, China). Juvenile hormone II and III were purchased from SciTech (Praha, Czech Republic) and Toronto Research Chemicals (Toronto, ON, Canada), respectively.

### 3.2. Insect

Larvae of the tobacco hornworm, *M. sexta*, were raised from eggs provided by Carolina Biological Supply Company (Burlington, NJ, USA) and reared on an artificial diet (BioServ) at 25 °C under a long-day (16 h light/8 h dark) photoperiod [15]. Pharate fifth instar larvae were set aside 4–7 h before lights off. The larvae molted within a few hours and were designated day 0. At the start of wandering, indicated by the appearance of a prominent dorsal vessel, the larvae were transferred to wooden blocks until pupation. Freshly ecdysed pupae were transferred to a wooden chamber containing a tobacco plant and 10% sucrose under a long-day photoperiod where the adult moths emerged. Moths were marked on the wings to distinguish the age of individuals [16].

### 3.3. Assays for JH Biosynthesis Assays In Vitro

On the day of adult emergence (day 0), females were marked and maintained in the wooden chamber until used. The assays for JH biosynthesis were performed using CA from day 3 females. A pair of CA with corpora cardiaca attached were incubated for 3 h at 30 °C in 100 µL of medium 199 (GIBCO) with Hanks’ salts, L-glutamine, 25 mM HEPES buffer (pH 7.2) and 2% Ficoll in the dark with gentle shaking. Compounds were dissolved in medium 199 for assay as described previously and used on the same day that the peptides were prepared. After incubation, 200 µl *n*-hexane containing 20 ng of citronellol as an internal standard was mixed with the incubation medium and the mixture was centrifuged at 2500× *g* for 5 min. The organic phases (upper layer) were removed and transferred to the analyzed vials. For *M. sexta*, it is known that JH II and JH III are predominantly made in adult (they contribute 99% of total JH, together) [17]. The change of total JH II and JH III titer was used to estimate the effects of JH biosynthesis in this study. The quantitative assay for JH II and JH III titer was determined by GC-MS/MS as described previously [18,19]. The retention time of JH II and JH III was 11.16 min and 10.36 min, respectively. The quantification transition for JH II was 107→105.4 (collsion energy: 45 eV). Its confirmation transitions were 81→79.6 (collsion energy: 40 eV), 121→119 (collsion energy: 40 eV), 121→104.9 (collsion energy: 15 eV) and 121→92.9 (collsion energy: 10 eV), respectively. For JH III, the quantification transition was 85.1→95.1 (collsion energy: 10 eV), the confirmation transitions were 81→79.1 (collsion energy: 5 eV), 94.9→67.1 (collsion energy: 10 eV), 120.9→93 (collsion energy: 10 eV) and 120.9→105.1 (collsion energy: 15 eV), respectively. Compounds were dissolved in medium 199 for assay and used on the same day that the peptides were prepared. Each data point on the dose-response figure represents replicate incubations of 9–13 experimental CA compared to control CA (i.e., no peptide added). EC_50_ (stimulator concentration_50_ defined as the concentration of stimulator that provokes a response halfway between the baseline and maximum response) values of Manse-AT and analogs were calculated from the dose-response curves.

Antagonistic effects of Manse-AT antagonists were measured with the compound mixture solutions (varying concentrations of the antagonist added together with Manse-AT) compared with the stimulation effects of Manse-AT. Dose response curves of antagonist on the inhibition of JH stimulation of Manse-AT was obtained using different concentrations of antagonist mixed with 1 nM Manse-AT (Supplementary Materials, Figure S1). Each data point on the dose-response figure was calculated using the Formula (1).

(R_Manse-AT_ − R_Mixture_)/(R_Manse-AT_ − R_Control_) (1)

R_Manse-AT_ is the average JH biosynthesis rate of 1 nM Manse-AT, R_Mixture_ is the rate of mixture contained different concentration antagonist and 1 nM Manse-AT, R_Control_ is the average rate of medium without any peptides.

Data presented as percentages were log-transformed before statistical analyses. Data were analysed using a one-way analysis of variance (ANOVA) with a Dunnett’s multiple comparison test as the post hoc determination of significance using Prism Graph Pad version 5.0 (GraphPad Software, San Diego, CA, USA). Dose-response curves were also prepared using the computer program GraphPad Prism. Values are expressed as mean ± standard errors (S.E.M.) with N indicating the number of samples measured.

### 3.4. Peptide Synthesis

Manse-AT analogs and antagonists were synthesized from Rink Amide-AM resin (198 mg, 0.1 mmol) using the standard Fmoc/tBu chemistry and HBTU/HOBt protocol. Incoming amino acids were activated with HOBt (41 mg, 0.3 mmol), HBTU (114 mg, 0.3 mmol) and DIEA (105 μL, 0.6 mmol) in DMF (5 mL) for 5 min, and couplings were run for 2 h. Removal of the *N*-terminal Fmoc group from the residues was accomplished with 20% piperidine in DMF (5 mL) for 20 min. The peptides were cleaved from the resin with TFA (10 mL) containing 8.4% phenol-dithioglycol (3:1), 4.3% thioanisole and 4.3% water for 2 h.

All the crude compounds were purified on a C_18_ reversed-phase preparation column with a flow rate of 10 mL/min using acetonitrile/water (50:50) containing 0.06% TFA as an ion-pairing reagent. UV detection was at 215 nm. The purity of each compound was greater than 95%. The structures of the analogs were confirmed by the presence of the following molecular ions using an 1100 series LC/MSD Trap (VL) (Agilent Technologies, Santa Clara, CA, USA). The structures of all target compounds are shown in Table 1 and Table 2.

### 3.5. Homology Search and Modeling

To identify the GPCR modeling template which most closely related to Manse-ATR (GenBank acc. No. HQ634154), we searched the Protein Data Bank database (PDB) using the BLASTP algorithm (version 2.2.28) [20] at the National Center for Biotechnology Information website with the protein sequence of Manse-ATR as the query sequence. We identified the nociceptin/orphanin FQ receptor and used the crystal structure of this receptor in complex with a peptide mimetic (PDB ID: 4EA3) as a template for homology modeling.

The file of the template was downloaded and all other unnecessary atoms were deleted except the atoms that matched the target. Homology modeling of Manse-ATR was carried out using FUGUE and ORCHESTRAR module in Sybyl-X 2.0. Energy minimization was performed using the minimize program in Sybyl-X 2.0 with steepest descent algorithm, AMBER7 FF99 as the force field and Gasteiger-Huckel as the atomic point charges [21]. The minimization was terminated when the RMS gradient convergence criterion of 0.05 kcal/(mol·Å) was reached. The qualities of these models were analyzed by PROCHECK [22].

### 3.6. Docking Calculations

The Surflex-Dock module implemented in the Sybyl program was used for the docking studies [23]. All the Manse-AT analogs were docked into Manse-ATR by an empirical scoring function and a patented search engine in Surflex-Dock. Protomol, a representation of a ligand making every potential interaction with the binding site, was applied to guide molecular docking. Protomols could be established by three manners: (1) Automatic: Surflex-Dock finds the largest cavity in the receptor protein; (2) Ligand: a ligand in the same coordinate space as the receptor; (3) Residues: specified residues in the receptor. In this study, the automatic docking was applied. Other parameters were established by default in the software. Surflex-Dock scores (total scores) were expressed in −lgK_d_ units to represent binding affinities.

To identify the binding pocket and accessibility of the pocket of Manse-ATR, Manse-AT (6–13) was used as the ligand. Blind docking calculations were carried out and the information of the pocket in the crystal structure of nociceptin/orphanin FQ receptor in complex with a peptide mimetic (PDB ID: 4EA3) was referenced during the docking calculations. The molecule was subjected to 30 trials of blind docking to search for the binding site. Once the binding site was identified, the protein-ligand complex with the highest total score was matched to the structure-activity relationship of AT analogs and used for further docking calculations.

Surflex-Dock Protomol was prepared using the Manse-AT (6–13) ligand inserted into the Manse-ATR model as above, with a threshold value of 0.5 and a Bloat of 0 Å. Surflex-Dock GeomX (SFXC) protocol was used, the search grid was expanded in 3 Å. Fifty additional starting conformations were used for each molecule and 30 conformations per fragment. Results were analyzed using the Sybyl program and the most stable pose which has the same pocket as the complex with Manse-AT (6–13) for each molecule was chosen as the preferred one inside the Manse-ATR.

### 3.7. Molecular Dynamics Simulations

The Manse-ATR in complex with Manse-AT (6–13) and analogs were used for performing MD simulations. The model of the 1-palmitoyl-2-oleoyl-*sn*-glycero-3-phosphatidylcholine (POPC) bilayer was used for simulation of the phospholipid environment [24]. Lipid molecules within 3 Å of the receptor were eliminated. The complexes were manually inserted into the center of the POPC bilayer. The 7TM helices were oriented approximately parallel to the hydrocarbon chains of the phospholipids, and the hydrophilic loops were placed into water layers. These were inserted into a water box (TIP3P [25] water model) with eliminating the water molecules within 3 Å of the receptor. These complexes were energy minimised, using the minimize program with the method Broyden-Fletcher-Goldfarb-Shanno (BFGS) in Sybyl-X 2.0. The force field was AMBER7 FF99 and the atomic point charge was Gasteiger-Huckel, for 500 steps to remove bad contacts. After initial minimization, the complexes were subjected to further optimization using the Dynamics program of Sybyl with the AMBER7 FF99 force field and Gasteiger-Huckel charges. The system setup for simulation included a 12 Å cutoff for non-bonded van der Waals interactions, and periodic boundary conditions. Constant temperature (300 K) and volume were maintained with time constant for heat bath coupling of 100 fs. The time step of 1 fs was used to integrate the equations of motion, and the snapshot time was 5 fs. The Boltzmann initial velocity was used to start the simulation. Other parameters were set by default in Sybyl. The system was equilibrated at 400 K for 0.1 ns followed by data collection, at regular intervals, for 10 ns. Each structure collected was subjected to 0.1 ns of simulated annealing to 300 K. The final 100 structures were energy minimized and clustered using cut-off distance of <0.2 nm.

## 4. Conclusions

Insect GPCRs provide an opportunity to develop highly effective and selective pesticides. However, the structures of insect GPCRs are not well characterized, and no crystal structure of an insect GPCR has been published. Discovery of new insecticides with insect neuropeptide GPCRs as the targets is still at the early stages. A target-based genome-to-lead approach for GPCRs as targets for next generation pesticides has been utilized to identify novel insecticidal molecules that disrupt GPCR-mediated processes in insects [26]. In this study, we gave a discovery of novel Manse-AT antagonist molecules with homology modeling, docking, molecular simulation and structure-activity relationship, which potential for pesticide development. Our predictive GPCR model will be useful for design or virtual screening of novel Manse-ATR agonist and antagonist. Manse-AT antagonists Manse-AT (10–13) and analogs have potential as insecticides for lepidopteran pest control.

## Figures and Tables

**Table 1:** Structures, stimulation of JH biosynthesis and free energy of binding of Manse-AT and analogs.

Peptide	Sequence	Stimulation of JH Biosynthesis		∆*G*_b_ (kJ/mol)
		EC_50_ (nM) ^a^ (95% CI ^b^)	Stimulation at 1 μM (Repeat Number)	
Manse-AT	GFKNVEMMTARGFa	0.9 (0.2–4.0)	426.6 ± 86.3 (9)	
Manse-AT (6–13)	EMMTARGFa	18.4 (4.8–70.5)	600.5 ± 98.5 (9)	−65.23
Manse-AT (7–13)	MMTARGFa	228.4 (53.0–985.0)	232.9 ± 33.7 (9)	−48.48
Manse-AT (8–13)	MTARGFa	2492 (1238–5017)	133.1 ± 22.3 (9)	−33.63
Manse-AT (9–13)	TARGFa	No effect	10.6 ± 21.0 (11)	−7.27
Manse-AT (10–13)	ARGFa	No effect	8.1± 17.6 (13)	3.23 ^c^
Manse-AT (11–13)	RGFa	No effect	8.3 ± 16.4 (9)	11.03
Manse-AT (6–13) [Ala^1^]	AMMTARGFa	393.7 (146.9–1055)	138.0 ± 12.7 (9)	−45.52
Manse-AT (6–13) [Ala^2^]	EAMTARGFa	691.6 (238.1–2009)	89.6 ± 14.6 (9)	−42.35
Manse-AT (6–13) [Ala^3^]	EMATARGFa	884.3 (288.0–2715)	91.0 ± 15.9 (9)	−40.32
Manse-AT (6–13) [Ala^4^]	EMMAARGFa	8958 (1803–44,500)	10.8 ± 6.5 (9)	−23.29
Manse-AT (6–13) [Ala^6^]	EMMTAAGFa	No effect	10.3 ± 7.8 (9)	10.76
Manse-AT (6–13) [Ala^7^]	EMMTARAFa	40.5 (13.9–118.7)	378.4 ± 49.8 (9)	−65.09
Manse-AT (6–13) [Ala^8^]	EMMTARGAa	No effect	4.5 ± 8.5 (9)	7.54

^a^ Repeat number of each concentration on the stimulation of JH biosynthesis is 9–13. ^b^ 95% CI: 95% Confidence Intervals. ^c^ ∆*G*_b_ value of Manse-AT (10–13) is 3.23 kJ/mol when it was docked into the same binding pocket of Manse-AT (6–13), but it can be docked into a different binding pocket of Manse-ATR with a negative ∆*G*_b_ value, −84.37 kJ/mol.

**Table 2:** Structures and antagonistic effects on JH biosynthesis of Manse-AT antagonists.

No.	Structure	Antagonistic Effects on JH Biosynthesis IC_50_ (nM) ^a^ (95% CI ^b^)
Manse-AT (10–13)	Ala-Arg-Gly-Phe-NH_2_	0.9 (0.2–5.4)
**1**	Gly-Arg-Gly-Phe-NH_2_	1.2 (0.4–3.8)
**2**	Leu-Arg-Gly-Phe-NH_2_	35.4 (15.9–78.6)
**3**	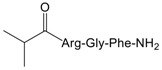	26.1 (8.8–78.1)
**4**	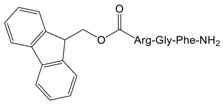	No effect
**5**	Ala-Ala-Gly-Phe-NH_2_	No effect
**6**	Ala-Leu-Gly-Phe-NH_2_	No effect
**7**	Ala-Arg-Ala-Phe-NH_2_	17.5 (8.3–37.1)
**8**	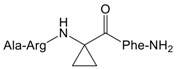	1031 (305.5–3479)
**9**	Ala-Arg-Gly-Ala-NH_2_	No effect
**10**	Ala-Arg-Gly-Tyr-NH_2_	1.0 (0.4–2.5)
**11**	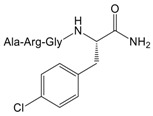	8.4 (3.9–18.3)
**12**	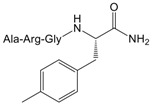	9.4 (3.2–27.1)
Manse-AT (11–13)	Arg-Gly-Phe-NH_2_	No effect

^a^ Repeat number of each concentration on the stimulation of JH biosynthesis is 9–13. ^b^ 95% CI: 95% Confidence Intervals.

## Supplementary Materials

The following are available online, Figure S1: Dose response curves of Manse-AT on JH biosynthesis; Figure S2: The −lgEC50 value (I) and the stimulation of JH biosynthesis at 1 μM (II) of truncated Manse-AT analogs for JH biosythesis; Figure S3: The stimulation of JH biosynthesis at 1 μM of alanine-replacement Manse-AT (6–13) analogs for JH biosythesis; Figure S4: Ramachandran plot of the 3D model of Manse-ATR.
