# Supplementary material for: Discovery of a Manduca sexta Allatotropin Antagonist from a Manduca sexta Allatotropin Receptor Homology Model
Source: Molecules. 2018 Apr 3;23(4):817. doi: 10.3390/molecules23040817 (PMC6017089; doi:10.3390/molecules23040817)
Supplement: Supplementary file 1 [file molecules-23-00817-s001.pdf]

Discovery of a *Manduca sexta* Allatotropin Antagonist from a *Manduca sexta* Allatotropin Receptor Homology Model

Zhen Peng Kai [1], Jing Jing Zhu [1,2], Xi Le Deng [3], Xin Ling Yang [3,*] and Shan Shan Chen [2,*]

1 School of Chemical and Environmental Engineering, Shanghai Institute of Technology, Shanghai 201418, China; kaizp@sit.edu.cn (Z.K.); 15212123252@163.com (J.Z.)

2 Institute of Agro-food Standards and Testing Technologies, Shanghai Academy of agricultural Science, Shanghai 201403, China

3 Department of Applied Chemistry, College of Science, China Agricultural University, Beijing 100193, China; chemdxl@gmail.com

* Correspondence: yangxl@cau.edu.cn (X.Y.); cssm100@163.com (S.C.); Tel.: +86-(0)10-6273-2223 (X.Y.); +86-(0)21-6713-1635 (S.C.)

Table of Contents

Supplementary Figures

Figure S1. Dose response curves of Manse-AT on JH biosynthesis.

Figure S2. The −lgEC50 value (I) and the stimulation of JH biosynthesis at 1 μM (II) of truncated Manse-AT analogs for JH biosythesis.

Figure S3. The stimulation of JH biosynthesis at 1 μM of alanine-replacement Manse-AT (6–13) analogs for JH biosythesis.

Figure S4. Ramachandran plot of the 3D model of Manse-ATR.

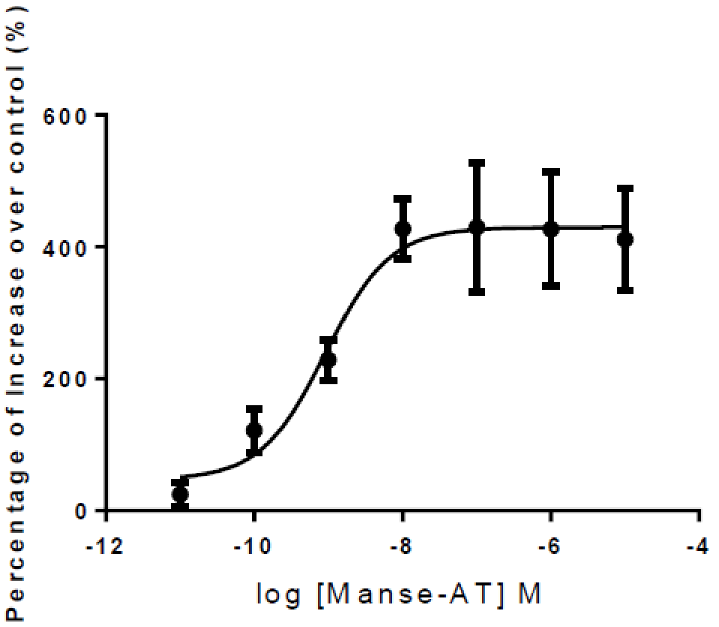

Figure S1. Dose response curves of Manse-AT on JH biosynthesis by CA from day 3 female *Manduca sexta*, in vitro.

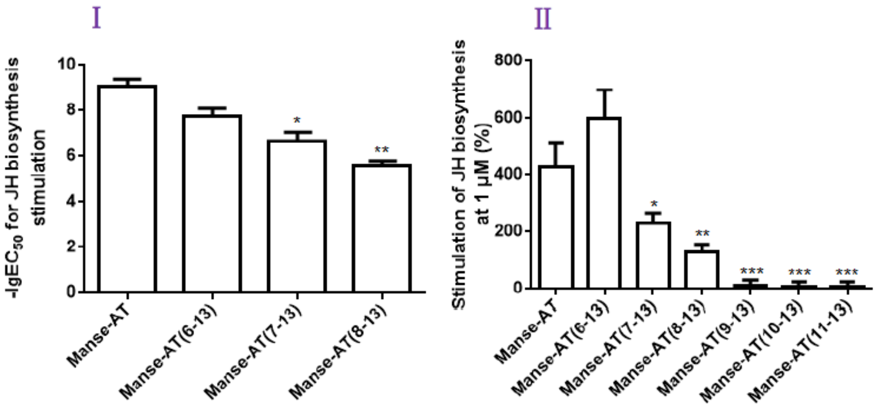

Figure S2. The $-lgEC_{50}$ value (I) and the stimulation of JH biosynthesis at 1 μm (II) of truncated Manse-AT analogs for JH biosysthesis. Asterisks indicate significant differences between Manse-AT and analogs as determined by Dunnett's multiple comparison test following one-way ANOVA: * $0.01 < P < 0.05$; ** $0.001 < P < 0.01$; *** $P < 0.0001$.

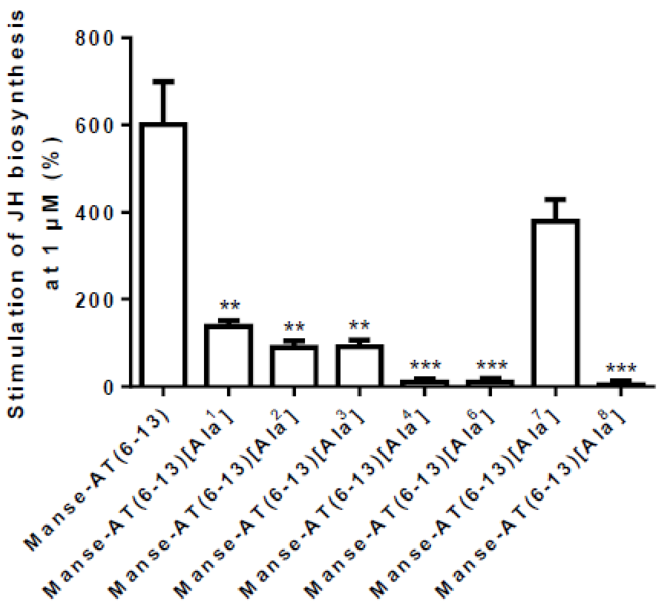

Figure S3. The stimulation of JH biosynthesis at 1 μM of alanine-replacement Manse-AT (6–13) analogs for JH biosysthesis. Asterisks indicate significant differences between Manse-AT (6–13) and analogs as determined by Dunnett's multiple comparison test following one-way ANOVA: ** $0.001 < P < 0.01$; *** $P < 0.0001$.

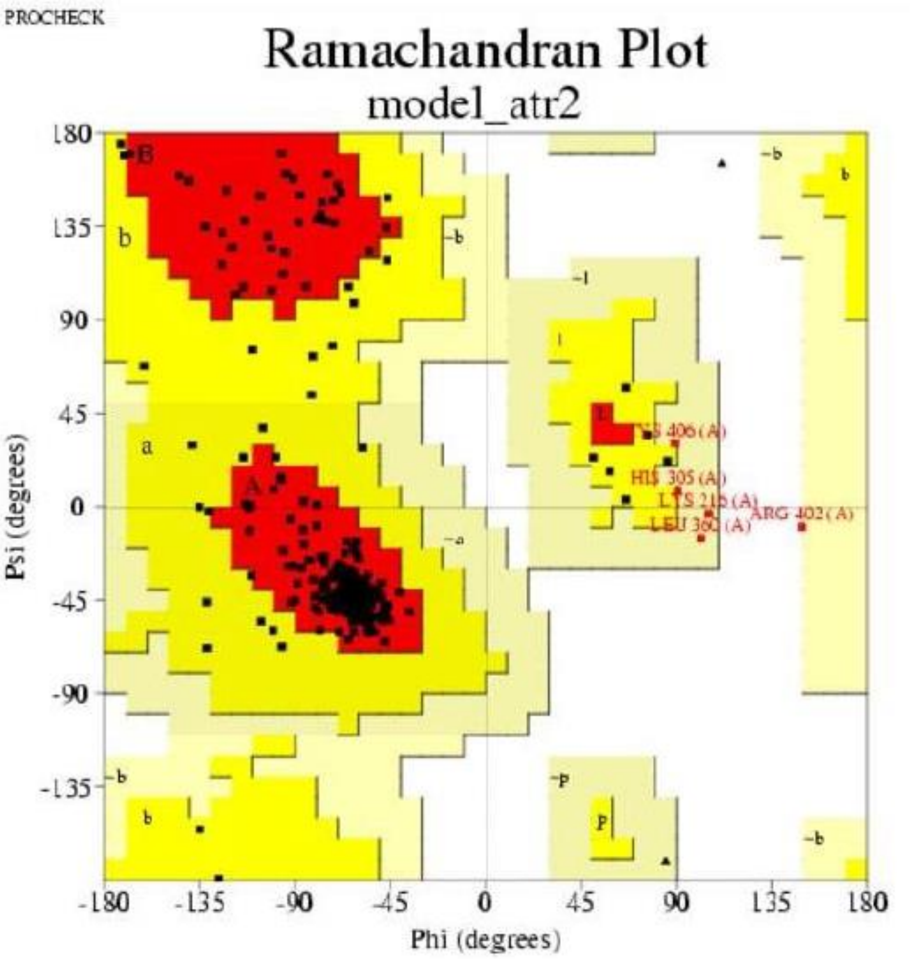

Figure S4. Ramachandran plot of the 3D model of Manse-ATR.
